# Saline–Alkaline Stress-Driven Rhizobacterial Community Restructuring and Alleviation of Stress by Indigenous PGPR in Alfalfa

**DOI:** 10.3390/plants14243844

**Published:** 2025-12-17

**Authors:** Min Wang, Ting Han, Fenghua Huang, Xiaochen Li, Jiayao Shan, Dongmei Zhang, Zhongbao Shen, Jianli Wang, Kun Qiao

**Affiliations:** 1 Institute of Agricultural Remote Sensing and Information, Heilongjiang Academy of Agricultural Sciences, Harbin 150086, China; wmfayewong@163.com (M.W.); hfh71@126.com (F.H.); lixiaochen1016@163.com (X.L.); hua4472hua@163.com (J.S.); 2 Grass and Science Institute of Heilongjiang Academy of Agricultural Sciences, Harbin 150086, China; zhd_mei@163.com; 3 College of Horticulture and Landscape Architecture, Northeast Agricultural University, Harbin 150030, China; 13294500270@163.com

**Keywords:** saline–alkaline stress, alfalfa, rhizosphere microbial community, microbial functional traits, plant growth-promoting rhizobacteria (PGPR)

## Abstract

Background: The Songnen Plain in China contains soda saline–alkaline soil, wherein salinity and alkalinity severely constrain crop productivity. Alfalfa (*Medicago sativa* L.) is a forage legume that has adapted to moderate saline–alkaline conditions, but how its rhizosphere microbial community facilitated this adaptation remains unclear. Methods: Using 16S rRNA gene sequencing, we compared alfalfa rhizosphere bacteria in saline–alkaline soil (AS) and control soil. Bacteria isolated from AS were screened for plant growth-promoting traits, with the most effective strains validated in pot experiments involving 50 mM NaHCO_3_. Results: Compared with the control soil bacterial community, the AS bacterial community was significantly enriched with Methylomirabilota and unclassified bacteria (phylum level), with the genus *RB41* identified as the most discriminative biomarker. Gene functions predicted using PICRUSt2 reflected the responsiveness of this community to environmental stressors. Inoculations with *Pseudomonas laurentiana* strain M73 and *Stenotrophomonas maltophilia* strain M81, which were isolated from AS, significantly improved alfalfa growth and health under NaHCO_3_ stress. Conclusions: Saline–alkaline conditions in the Songnen Plain reshape the alfalfa rhizosphere bacterial community, enriching for specific taxa and potentially enhancing microbial functions associated with stress resistance. Strains M73 and M81 can effectively promote stress tolerance, making them useful microbial resources for improving soil conditions.

## 1. Introduction

Soil salinization is a critical issue with ecological and agricultural implications worldwide, and it affects more areas each year. Last year, it affected 1.381 billion hectares of land, resulting in crop yield losses of approximately 0.5% [1]. Among these regions, the Songnen Plain in Northeast China is characterized by typical soda saline–alkaline soils. The combined effects of salinity and alkalinity on plants severely inhibits the growth of most crops, which in turn constrains agricultural production [2].

Previous research showed that rice (*Oryza sativa* L.) is particularly sensitive to saline–alkaline conditions during the seedling and reproductive stages, resulting in inhibited growth and decreased yield [3,4,5]. Maize (*Zea mays* L.) seedlings also exhibit significantly suppressed growth and disrupted ion homeostasis in saline–alkaline environments [6,7]. Wheat (*Triticum aestivum* L.) is most sensitive to saline–alkaline stress at the seedling stage, with alkaline conditions severely altering the water content, spectral characteristics, and growth [8]. Other crops are similarly affected by saline–alkaline stress, resulting in the excessive accumulation of reactive oxygen species (ROS), decreased photosynthetic and root activities, osmotic imbalance, and ion toxicity. Notably, the combined exposure to salinity and alkalinity causes substantially more damage than exposure to salinity alone [9,10,11,12].

Certain plant species exhibit varying degrees of tolerance to saline–alkaline stress, with some even capable of surviving in moderately to severely saline–alkaline soil [13,14,15]. Among these, alfalfa (*Medicago sativa* L.) is a high-quality protein-rich forage crop that is not only able to thrive in mildly to moderately saline–alkaline soils, but is also able to fix nitrogen, tolerate drought stress, and endure poor soil conditions [16,17,18]. Previous studies have demonstrated that alfalfa is able to enhance soil nutrient content and decrease salinity, while also increasing soil microorganism abundance and diversity [19,20,21,22]. These combined effects improve the soil ecosystem to make it more favorable for plant growth. Thus, alfalfa is a promising candidate for the bioremediation of saline–alkaline soils. Moreover, cultivating alfalfa on saline–alkaline land may help restore arable farmland and increase the utility of marginal soils, potentially leading to ecological and financial benefits. However, extreme saline–alkaline conditions can adversely affect alfalfa (e.g., suppressed seed germination, inhibited photosynthesis, leaf wilting, and even death), which may severely restrict its applicability for saline–alkaline soil remediation [23,24,25]. Therefore, systematically exploring effective strategies for enhancing the tolerance of alfalfa to saline–alkaline environments is critical for ensuring stable crop production and ecological restoration in high-stress environments.

Under natural conditions, plants are exposed to various biotic and abiotic stresses, including drought, salinity, pests and diseases, and extreme temperatures. However, the rhizosphere microbiome, which may be considered as a “second genome” for plants, plays a crucial role in rapidly responding to environmental challenges and regulating plant growth and development [26]. Prior research has established clear correlations between specific plant growth-promoting rhizobacteria (PGPR) and plant salt tolerance. The relevant microbial groups, including arbuscular mycorrhizal fungi [27,28], *Enterobacter* spp. [29], *Bacillus* spp. [30], and *Pseudomonas* spp. [31], can effectively enhance plant salt tolerance through various physiological and biochemical mechanisms. For example, inoculating sunflower (*Helianthus annuus* L.) plants with *Pseudomonas entomophila* PE3 alleviates the effects of salt stress because the bacterium secretes exopolysaccharides that sequester sodium ions and mitigate oxidative damage, while also enhancing plant growth by promoting phytohormone production and improving nutrient solubilization [32]. A previous study on *Pseudomonas chlororaphis* revealed that bacterial phenazines can enhance wheat salt tolerance by decreasing leaf ROS accumulation and increasing antioxidant enzyme activities, thereby mitigating oxidative damage [33]. Furthermore, *Enterobacter ludwigii* B30 enhances bermudagrass (*Cynodon dactylon *(L.) Persoon) salt tolerance by increasing antioxidant enzyme activities and maintaining ion homeostasis (K^+^/Na^+^), while also modulating the rhizosphere microbiome to produce a beneficial microbial community [34]. Given this context, a key question is whether alfalfa similarly leverages its rhizosphere microbes for its adaptation to saline conditions. Preliminary evidence suggests this is the case. Specifically, *Bacillus subtilis* ssp. *subtilis* NRCB002 and *B. subtilis* NRCB003, which were isolated from the alfalfa rhizosphere, positively affect alfalfa growth and salt tolerance [35]. Moreover, salt-tolerant alfalfa cultivars recruit a greater abundance of beneficial PGPR in their rhizosphere than salt-sensitive varieties, leading to normal growth under stress conditions [36]. These findings suggest that plants with varying levels of salt tolerance differentially recruit distinct microbial communities (composition and abundance) in response to salt stress. However, the response dynamics of alfalfa rhizosphere bacteria to soda saline–alkaline stress, including compositional shifts and functional adaptations, remain poorly characterized. Furthermore, identifying specific stress-ameliorating microbial taxa and their mechanisms that improve alfalfa tolerance to saline–alkaline conditions depends on systematic investigations.

The application of PGPR represents a key biological strategy for enhancing plant adaptations to saline–alkaline soils [37,38,39]. Excessively high salt concentrations can inhibit the growth of certain PGPR, with salt tolerance being a key factor determining their efficacy [40]. Therefore, the capacity to mitigate salt stress represents an essential criterion for screening PGPR strains [41]. Accordingly, when screening PGPR for their utility in soda saline–alkaline environments, strain tolerance to alkaline salts must be evaluated. In addition, inoculating plants with indigenous microorganisms, particularly rhizosphere microbes, can alleviate the detrimental effects of salt stress [42,43]. Hence, indigenous PGPR strains derived from soda saline–alkaline environments may be useful as microbial inoculants for enhancing alfalfa stress tolerance. Accordingly, alfalfa cultivar ‘Nongjing 1’, which is tolerant to the saline–alkaline conditions in the Songnen Plain, was used in this study. The study objectives were to: (1) characterize the structural shifts in the rhizosphere bacterial community under saline–alkaline conditions via 16S rRNA gene sequencing; (2) predict the potential functional changes within this community; and (3) isolate, screen, and evaluate indigenous PGPR from this environment for their efficacy in enhancing alfalfa tolerance to saline–alkaline conditions. This study may provide theoretical insights into plant–microbe interactions in saline–alkaline soils as well as microbial resources relevant to sustainable agricultural development in the Songnen Plain.

## 2. Results

### 2.1. Experimental Design and Overview

We conducted a comprehensive study to investigate the response of the alfalfa rhizosphere bacterial community to saline–alkaline stress and to screen for beneficial indigenous PGPR. Soil samples were collected from the rhizosphere of saline–alkaline stress-tolerant alfalfa cultivar ‘Nongjing 1’ grown in natural saline–alkaline soil (AS) and control soil (CK) in the Songnen Plain. Some AS samples were used for (1) 16S rRNA gene sequencing to characterize the microbial community and (2) isolating culturable bacteria. Isolated bacteria were subsequently screened for plant growth-promoting (PGP) traits, with the most promising strains (including single strains and a composite strain, FH) validated regarding efficacy. Pot experiments and germination tests were conducted using 50 mM NaHCO_3_ to simulate stress to quantitatively assess the ability of these selected PGPR to enhance alfalfa tolerance to saline–alkaline conditions.

### 2.2. Soil Physicochemical Properties

An analysis of soil basic properties revealed clear differences between AS and CK environments, which were important for interpreting microbial responses. As expected, AS was characterized by a higher pH than CK, reflecting its saline–alkaline conditions, as well as a high nutrient content. Total nitrogen, available nitrogen, and soil organic matter (SOM) contents were 52.4%, 64.4%, and 54.9% higher, respectively, in AS than in CK (Table 1).

### 2.3. Changes in the Microbial Community Composition and Structure of the Alfalfa Rhizosphere Under Saline–Alkaline Conditions

**Figure 1:**
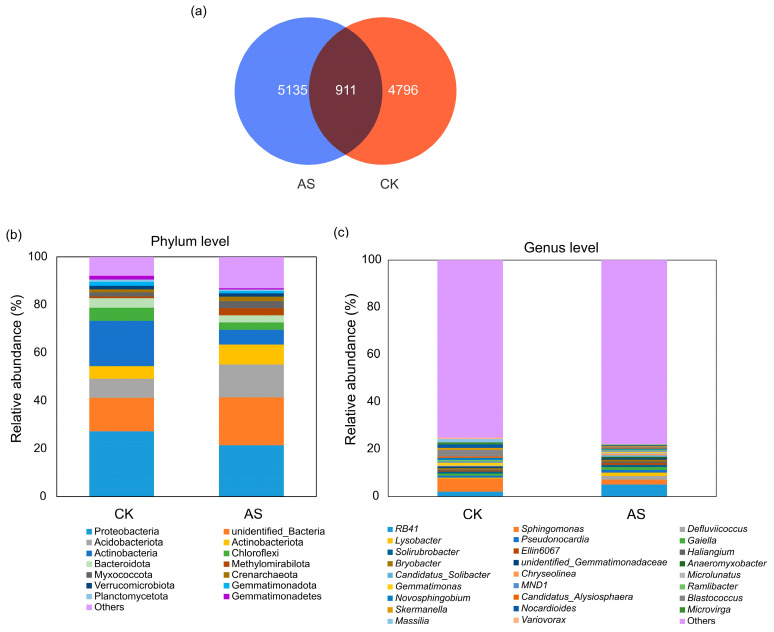
Differences in the rhizosphere bacterial community composition between saline–alkaline soil (AS) and control soil (CK). (**a**) Venn diagram of OTUs (97% similarity) showing the number of unique and shared OTUs in AS (*n* = 3) and CK (*n* = 3). (**b**) Bacterial community composition at the phylum level. Only phyla with a relative abundance ≥1% in at least one group are shown; the rest are grouped as ‘Others’. (**c**) Bacterial community composition at the genus level. Genera with a relative abundance ≥ 0.5% in at least one group are presented, but most were classified as ‘Others’ (relative abundance < 0.5%).

On the basis of 16S rRNA gene sequencing of three independent biological replicates per group (AS and CK), 22,607 operational taxonomic units (OTUs) were obtained for rhizosphere bacterial communities. AS and CK groups contained 6046 and 5707 OTUs, respectively; 911 OTUs were common to both groups (Figure 1a).

A total of 60 bacterial phyla were identified, among which the following 11 were dominant (relative abundance ≥ 1%) in AS: Proteobacteria, unidentified_Bacteria, Acidobacteriota, Actinobacteriota, Actinobacteria, Chloroflexi, Bacteroidota, Methylomirabilota, Myxococcota, Crenarchaeota, and Verrucomicrobiota. By contrast, the following 13 phyla were dominant in CK: Proteobacteria, Actinobacteria, unidentified_Bacteria, Acidobacteriota, Chloroflexi, Actinobacteriota, Bacteroidota, Myxococcota, Gemmatimonadota, Gemmatimonadetes, Verrucomicrobiota, Crenarchaeota, and Planctomycetota (Figure 1b). Notably, relative abundances of Proteobacteria (21.43%), unidentified_Bacteria (20.00%), and Acidobacteriota (13.77%) were highest in AS. In CK, the most abundant phyla were Proteobacteria (27.27%), Actinobacteria (18.77%), and unidentified_Bacteria (13.93%).

A total of 472 bacterial genera were identified. The community was highly diverse, with no single genus dominating, as indicated by the large proportion (>75%) of genera classified in the ‘Others’ group (i.e., genera with a relative abundance < 0.5%; Figure 1c). The most abundant identifiable genera (relative abundance ≥ 0.5%) in the AS group were *RB41*, *Sphingomonas*, *Defluviicoccus*, *Lysobacter*, and *Pseudonocardia*, whereas *Sphingomonas*, *Blastococcus*, *RB41*, *Nocardioides*, *Massilia*, and *Gemmatimonas* were the most abundant genera in the CK group (Figure 1c). The relative abundance of all genera exceeding the 0.5% threshold in the AS and CK groups is detailed in Supplementary Figure S1. Although the overall community structure was determined, it remained unclear which taxa were significantly enriched by treatments. Therefore, we conducted Linear discriminant analysis Effect Size (LEfSe) analysis to identify differentially abundant genera.

### 2.4. Analysis of Microbial Diversity in the Alfalfa Rhizosphere Soil Under Saline–Alkaline Conditions

**Figure 2:**
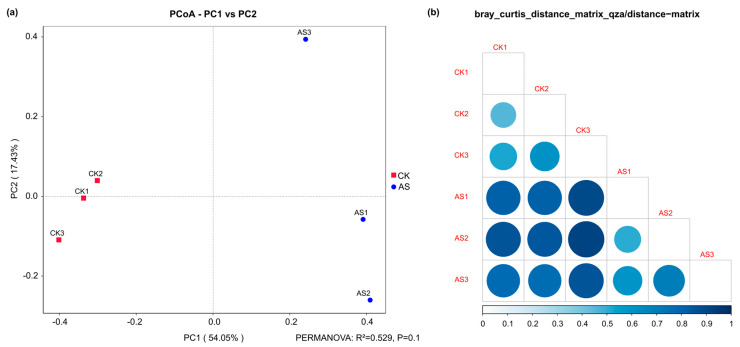
Differences in the bacterial community structure (beta-diversity) between saline–alkaline (AS) and control (CK) groups. (**a**) Principal coordinate analysis (PCoA) plot based on Bray–Curtis distances. AS and CK samples are clearly separated along PC1 (54.05% of the variance), suggesting that the saline–alkaline treatment was the primary factor shaping the bacterial community structure. AS samples were more dispersed than CK samples. (**b**) Heatmap of the Bray–Curtis dissimilarity matrix. Dark and large blue circles represent high dissimilarity between two samples, reflecting stronger within-group similarity (homogeneity) in the CK group than in the AS group. CK1–3: CK group samples; AS1–3: AS group samples.

An analysis of alpha-diversity revealed that both Chao 1 and Shannon indices were lower in the AS group than in the CK group, whereas the Simpson index was higher in the AS group than in the CK group; however, these differences were not significant (Figure S2). Despite the lack of significant differences in species richness and evenness (alpha-diversity), the bacterial community composition (beta-diversity) was markedly influenced by saline–alkaline conditions. More specifically, the overall community structure of AS was clearly separated from that of CK by a principal coordinate analysis (PCoA) (Figure 2a). This separation was corroborated by a Bray–Curtis dissimilarity heatmap, which revealed a relatively high within-group heterogeneity in the AS community, which was in contrast to the relatively homogeneous CK community (Figure 2b). According to PERMANOVA, the substantial effect of saline–alkaline conditions explained 52.9% of the community variation, although this model was not statistically significant at the conventional level (R^2^ = 0.529, *p* = 0.1) (Table S1).

### 2.5. Differentially Abundant Microbial Taxa Under Saline–Alkaline Conditions

**Figure 3:**
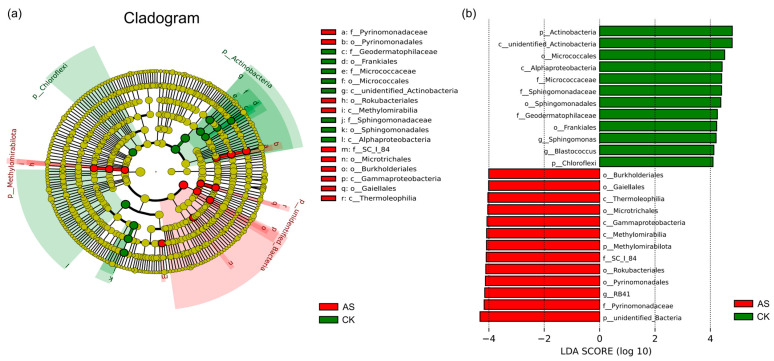
Evolutionary branching diagrams for the LEfSe analysis of rhizosphere bacterial communities under saline–alkaline conditions. (**a**) In the cladogram, concentric circles represent taxonomic levels (phylum to genus/species). Circle size is proportional to relative abundance. Yellow = non-significant; Red/Green = biomarkers for respective groups. Species abbreviations are indicated. (**b**) LDA score distribution (threshold > 4). Bar length = effect size (LDA score).

An examination of genus-level differences detected significant shifts in rhizosphere microbial community compositions in saline–alkaline soils. According to a *t*-test (*p* < 0.05), 25 distinct differentially abundant genera were identified, with nine genera significantly enriched in AS and 16 genera dominant in CK (Figure S3). A LEfSe analysis identified significant microbial biomarkers for distinguishing between AS and CK rhizosphere microbiomes. A hierarchical cladogram indicated that AS biomarkers (red nodes) were predominantly distributed in the phyla Methylomirabilota and unidentified_Bacteria (Figure 3a). Notably, Pyrinomonadaceae (family level) and Pyrinomonadales (order level) were both identified as AS-specific biomarkers, suggesting that these taxa are highly adaptable to saline–alkaline environments (Figure 3a). Interestingly, within the unclassified bacterial phylum (unidentified_Bacteria), two classes (Gammaproteobacteria and Thermoleophilia) and three orders (Microtrichales, Burkholderiales, and Gaiellales) were significantly enriched in AS (Figure 3a). This implies that despite their ambiguous taxonomic status, these unidentified bacteria may play critical roles in plant responses to soda saline–alkaline stress.

The most effective microbial biomarkers for discriminating between the two groups were determined (Figure 3b; LDA score > 4, *p* < 0.05). The AS group was dominated by *RB41* from Acidobacteriota (LDA = 4.1), whereas the CK group was characterized by *Sphingomonas* from Proteobacteria (LDA = 4.2) and *Blastococcus* from Actinobacteria (LDA = 4.1), which was consistent with *t*-test results (Figure S3).

### 2.6. Predicted Functions of Soil Bacteria

**Figure 4:**
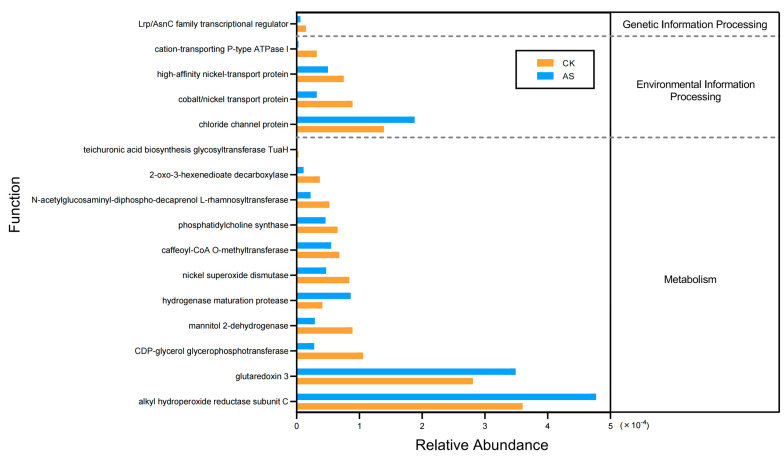
Differences in the abundance of bacterial genes with predicted functions between saline–alkaline soil (AS) and control soil (CK). Relative abundances of genes encoding specific stress-related proteins are presented; genes are grouped under their respective broad functional categories (e.g., Metabolism) as predicted by PICRUSt2.

PICRUSt2 revealed that saline–alkaline environmental conditions significantly altered microbial community functions (Figure 4). Specifically, genes encoding peroxiredoxin C subunit, glutathione reductase, and a chloride channel protein were more abundant in the AS group than in the CK group. By contrast, mannitol dehydrogenase, caffeoyl-CoA O-methyltransferase, and L-rhamnosyltransferase genes were significantly enriched in the CK group (Figure 4). Notably, the observed differences in the abundance of genes associated with nickel transport-related activities may imply that saline–alkaline conditions altered the bioavailability of metal ions (Figure 4).

### 2.7. Isolation and Phylogenetic Analysis of Culturable Microbes

**Figure 5:**
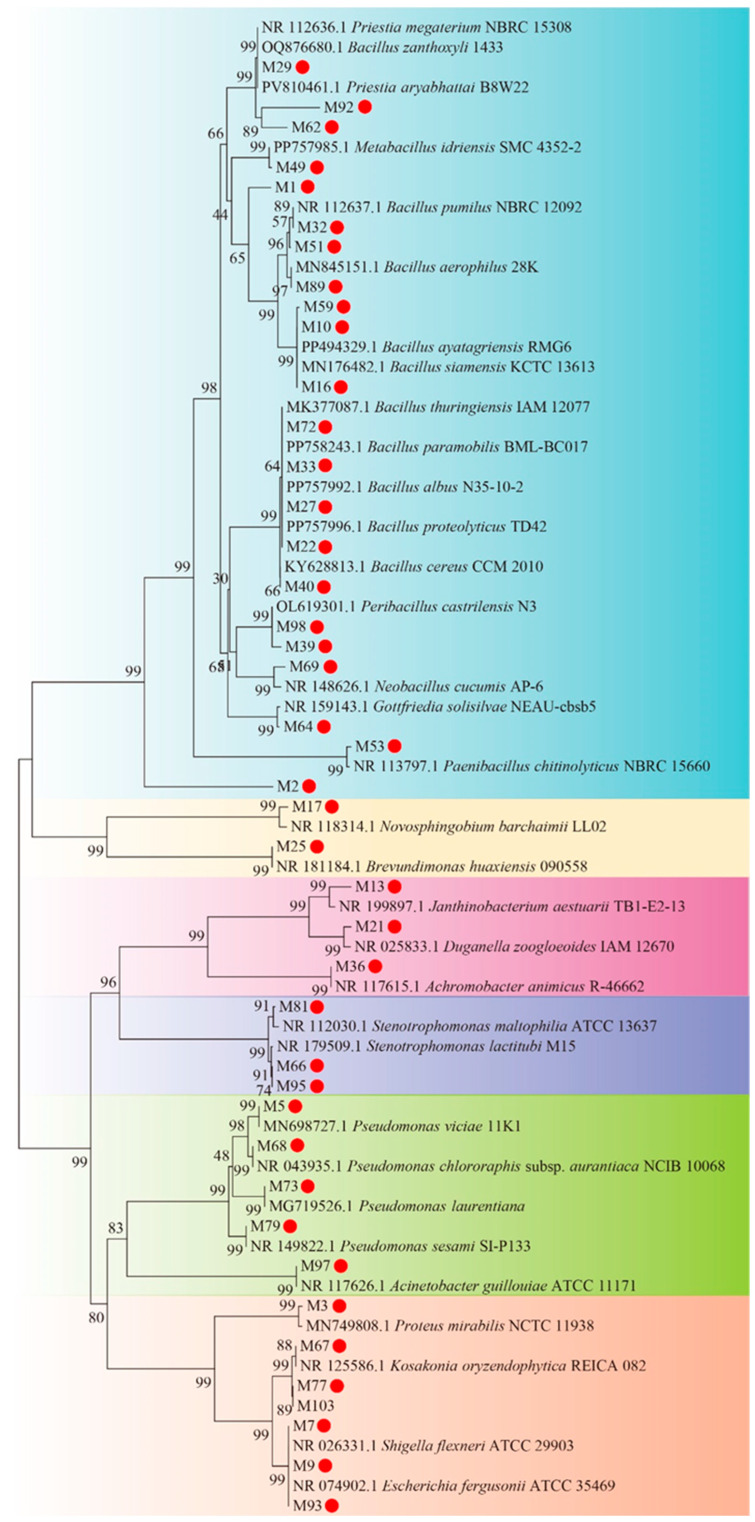
Phylogenetic tree constructed on the basis of 16S rRNA gene sequencing and the neighbor-joining method using MEGA7. Specifically, the Kimura 2-parameter substitution model was used, with 1000 bootstrap replicates. The scale bar indicates 0.01 substitutions per site. Red solid circles indicate microbial isolates from saline–alkaline soil.

A total of 42 microbial isolates were obtained from saline–alkaline soil samples using Reasoner’s 2A agar (R2A), 0.1× tryptic soy agar (TSA), and 0.1× lysogeny broth (LB) media under aerobic conditions. Taxonomic assignments on the basis of 16S rRNA gene sequencing revealed that these isolates were from 13 genera, with *Bacillus* (47.62%) as the dominant taxon (Table 2). A neighbor-joining phylogenetic tree further demonstrated the evolutionary relationships between isolates and reference strains (Figure 5), with 29 strongly supported nodes (bootstrap ≥ 70%). In the phylogenetic tree, M29 was clustered close to *Priestia megaterium* (bootstrap = 99), M73 was grouped close to *Pseudomonas laurentiana* (bootstrap = 99), and M81 was positioned close to *Stenotrophomonas maltophilia* (bootstrap = 91).

### 2.8. In Vitro Screening of PGP Traits

An analysis of 42 strains exposed to saline–alkaline conditions showed that most strains grew normally when treated with 50 and 100 mM NaHCO_3_, but the growth of some strains was inhibited. Following the treatment with 200 mM NaHCO_3_, only a few strains were able to grow, including M10, M27, M32, and M97 (Figure S4). Additionally, the inhibitory effect of NaCl on the growth of the analyzed strains increased as the NaCl concentration increased (i.e., relatively small colonies and decreased growth rate). Treatments with 700 and 1000 mM NaCl severely affected the growth of most strains, with the exception of M7, M29, M32, M62, M67, M73, M77, M89, and M92, which grew relatively slowly (Figure S5).

A total of 37 strains grew normally during a qualitative analysis of their nitrogen-fixing capacity (Figure S6). Additionally, among the 14 strains with a significant increase in the ammonium nitrogen content, *Bacillus* sp. M59, *P. laurentiana* M73, and *P. megaterium* M29 secreted relatively large amounts of extracellular ammonium nitrogen, reflecting strong nitrogen fixation, with ammonium nitrogen contents reaching 8.710, 7.604, and 5.051 mg/L, respectively (Figure S7). While screening for soil phosphate-solubilizing bacteria, 17 of the 42 tested strains (40.48%) were observed to exhibit phosphate-solubilizing activity, with relatively high activities detected for strains M2, M9, M7, M29, M95, M97, M81, M16, M27, and M73 (Figure S8). Indole-3-acetic acid (IAA) secretion by PGPR strains was assessed using a colorimetry-based method. Of the 42 analyzed strains, 28 (66.67%) secreted IAA, among which strains M9, M81, M95, M97, M25, M13, M89, and M59 produced relatively large amounts of IAA (123.09, 114.91, 104.06, 100.44, 97.65, 97.35, 97.35, and 94.91 mg/L, respectively; Figure S9).

**Figure 6:**
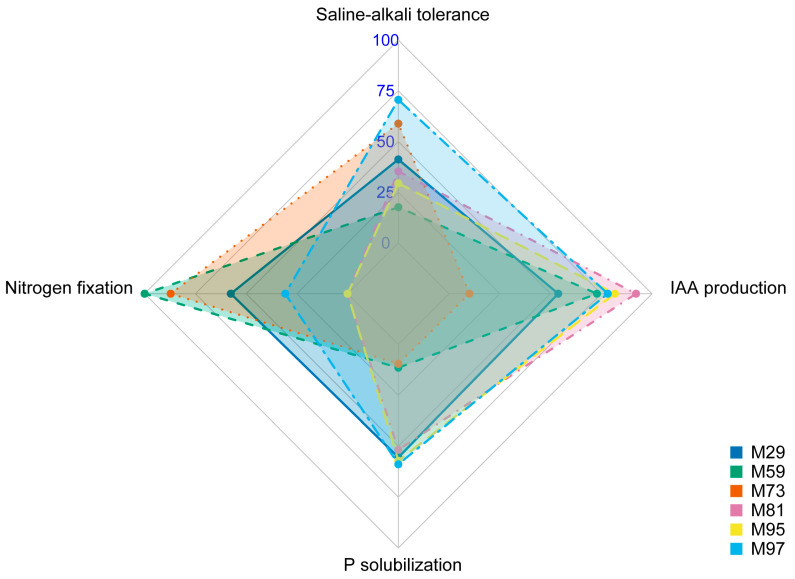
Multifunctional profiling of elite PGPR strains (normalized to 0–100%). Bacterial strains are differentiated by color: *P. megaterium* M29 (blue), *Bacillus* sp. M59 (teal), *P. laurentiana* M73 (orange), *S. maltophilia* M81 (pink), *Stenotrophomonas* sp. M95 (yellow), and *A. guillouiae* M97 (light blue).

On the basis of the comprehensive evaluation of 42 strains, six representative strains (from four genera: *Bacillus*, *Pseudomonas*, *Stenotrophomonas*, and *Acinetobacter*) ranked among the top 10 in terms of their resistance to NaHCO_3_ and NaCl in media as well as their ability to fix nitrogen, solubilize phosphate, and produce IAA were selected for a multifunctional analysis. Strains were grouped into the following three function-based categories (Figure 6): (1) broad-spectrum strains *Acinetobacter guillouiae* M97 and *P. megaterium* M29, which had a balanced performance (i.e., saline–alkaline resistance, nitrogen fixation, phosphate solubilization, and IAA production); (2) specialized strains *Bacillus* sp. M59, which had extremely high nitrogen-fixing activities but weak saline–alkaline resistance and phosphate-solubilizing ability, and *S. maltophilia* M81, which was able to produce large amounts of IAA; (3) intermediate strains *P. laurentiana* M73 and *Stenotrophomonas* sp. M95 with a moderate performance (two or three activities). Full data for all isolates are provided in Table S2.

### 2.9. Plant Growth-Promoting Effects of Selected Strains in Pot Experiments

**Figure 7:**
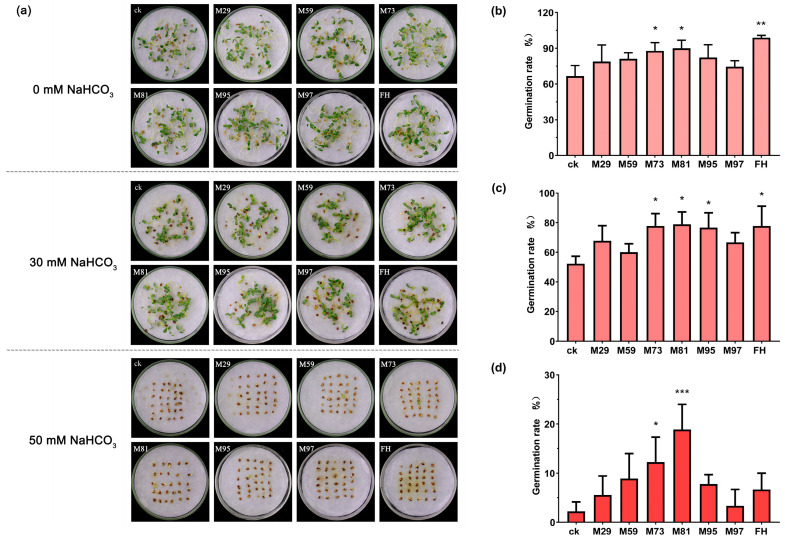
Effects of PGPR on alfalfa seed germination under different NaHCO_3_ conditions. (**a**) Germination of alfalfa seeds pre-treated with PGPR for 2 h in the presence of 0, 30, and 50 mM NaHCO_3_. Time-course analysis of germination rates (mean ± SD, *n* = 3) at (**b**) 0 mM, (**c**) 30 mM, and (**d**) 50 mM NaHCO_3_; ck indicates the control group without bacterial inoculation (sterile water added instead); FH represents a synthetic microbial consortium comprising six bacterial strains in equal proportions. Data were analyzed by a one-way ANOVA (* *p* < 0.05, ** 0.01 < *p* < 0.05, *** *p* < 0.01).

Germination assays demonstrated that the alfalfa seed germination rate decreased as the NaHCO_3_ concentration increased. A comparison with the non-inoculated control group indicated that inoculations with single-strain suspensions (i.e., six selected strains) and the FH suspension increased alfalfa seed germination rates (Figure 7a). Specifically, under NaHCO_3_-free conditions, the FH treatment and *S. maltophilia* M81 treatment significantly increased alfalfa germination rates relative to the control germination rate (i.e., no bacterial inoculation). Furthermore, when the NaHCO_3_ concentration was increased to 30 mM, the FH treatment and M81 treatment still significantly promoted germination (Figure 7b, *p* < 0.05). Notably, at 50 mM NaHCO_3_, only the *S. maltophilia* M81 treatment significantly increased the alfalfa seed germination rate (Figure 7b, *p* < 0.05).

**Figure 8:**
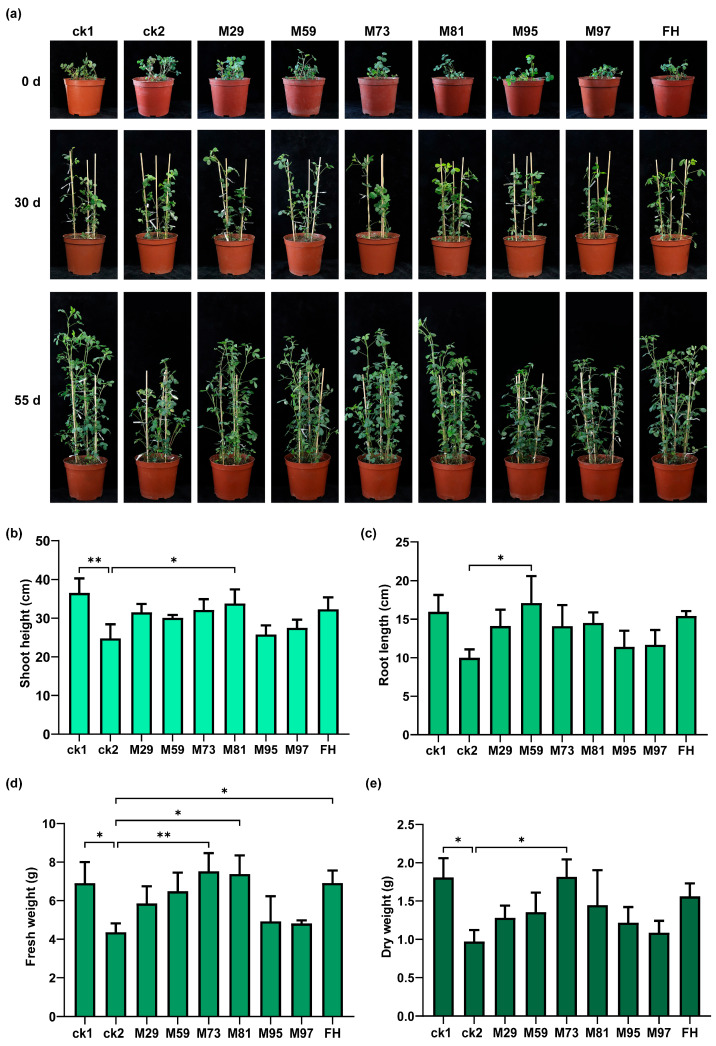
Effects of PGPR on alfalfa growth in the presence of NaHCO_3_. (**a**) Phenotypic characteristics, (**b**) shoot height, (**c**) root length, (**d**) fresh weight, and (**e**) dry weight; ck1: 50 mL water every 5 days + 6 mL water every 3 days; ck2: 50 mL 50 mM NaHCO_3_ every 5 days + 6 mL water every 3 days. Saline–alkaline conditions + bacterial inoculations (strains M29, M59, M73, M81, M95, M97, and FH): 50 mL 50 mM NaHCO_3_ every 5 days + 6 mL [specific bacterial strain] suspension every 3 days. Significant differences (* *p* < 0.05, ** 0.01 < *p* < 0.05) compared with ck2 (mean ± SD, *n* = 3; one-way ANOVA) are indicated.

Alfalfa growth performance was assessed following a 55-day co-cultivation with PGPR in the presence of NaHCO_3_ (Figure 8a). Saline–alkaline conditions severely impaired the above-ground growth of alfalfa plants, but inoculating with PGPR strains mitigated the inhibitory effect of NaHCO_3_ on seedling development. Specifically, alfalfa inoculated with *S. maltophilia* M81 were significantly taller than ck2 plants (*p* < 0.05), but they did not differ significantly from ck1 plants (non-saline–alkaline conditions) in terms of height (*p* > 0.05). Hence, *S. maltophilia* M81 effectively alleviated the suppressive effect of NaHCO_3_ on plant height (Figure 8b). In addition, plants inoculated with *Bacillus* sp. M59 had significantly longer roots than ck2 plants, with no significant difference from the root length of ck1 plants, suggesting that this strain effectively relieved the inhibitory effect of NaHCO_3_ on root elongation (Figure 8c). Moreover, fresh weights were significantly higher for plants inoculated with *P. laurentiana* M73, *S. maltophilia* M81, or FH than for ck2 plants (*p* < 0.05). Furthermore, the dry weight of M73-inoculated plants was significantly greater than that of ck2 plants (*p* < 0.05). These findings suggest that these strains can alleviate the inhibitory effects of NaHCO_3_ on plant biomass accumulation (Figure 8d,e).

**Figure 9:**
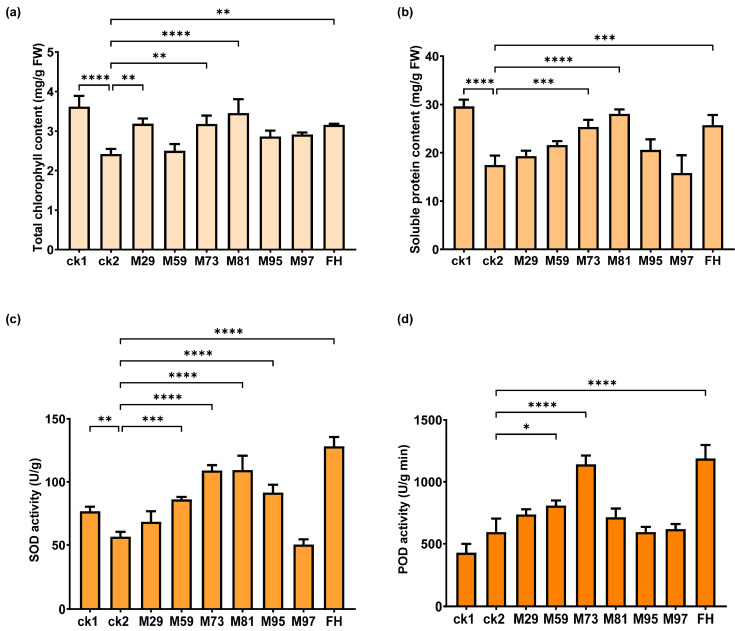
Effects of PGPR on physiological and stress resistance-related characteristics of alfalfa treated with NaHCO_3_. (**a**) Total chlorophyll content. (**b**) Soluble protein content. (**c**) SOD activity. (**d**) POD activity. SOD (WST-1 method) and POD activities were measured using assay kits from Beijing Solarbio Science & Technology Co., Ltd. (Beijing, China). Data (mean ± SD, *n* = 3) were analyzed by a one-way ANOVA (* *p* < 0.05, ** 0.01 < *p* < 0.05, *** 0.001 < *p* < 0.01, **** *p* < 0.001).

Consistent with the results presented in Figure 8, the inoculation with *P. laurentiana* M73 significantly increased chlorophyll and soluble protein contents as well as superoxide dismutase (SOD) and peroxidase (POD) activities (Figure 9). Strains *P. megaterium* M29, *Bacillus* sp. M59, *S. maltophilia* M81, and *Stenotrophomonas* sp. M95 also had considerable positive effects. Notably, FH significantly increased these physiological and antioxidant parameters of plants treated with NaHCO_3_, with a particularly pronounced effect on antioxidant enzyme activities (Figure 9).

## 3. Discussion

Rhizosphere bacteria play a key role in plant growth and development, nutrient acquisition, ecosystem functions, and tolerance to both biotic and abiotic stresses [44]. Profiling the alfalfa rhizosphere microbiome under saline–alkaline conditions is critical for elucidating stress response mechanisms, with possible implications for improving the tolerance of alfalfa plants to saline–alkaline environments. In this study, a comparative analysis revealed differences in the bacterial communities of AS and CK. At the phylum level, stress conditions in AS resulted in the development of a distinct, streamlined consortium dominated by 10 phyla, rather than the 12 phyla in CK (Figure 1b). Stress-tolerant phyla, such as Proteobacteria, Acidobacteriota, and Actinobacteriota, were significantly enriched in AS. These findings were in line with the results of previous studies on the dominant bacterial phyla in saline–alkaline soils [45,46,47], implying that Proteobacteria, Acidobacteriota, and Actinobacteriota are the most abundant and relatively active members of the bacterial community in saline–alkaline environments. Acidobacteriota may enhance plant adaptations to saline–alkaline conditions by increasing SOM contents through the decomposition of plant residues [48]. This is supported by a previous study in which the abundance of Acidobacteriota increased significantly when wheat was cultivated in saline–alkaline soil [49]. This community shift was further corroborated at the genus level, with key taxa (e.g., *RB41*) enriched in AS, while *Sphingomonas* and *Blastococcus* were markedly more abundant in CK (Figure 3b). The dominance of *RB41* in saline–alkaline soil is consistent with the results of previous research [50,51]. Another study demonstrated that *RB41* abundance is positively correlated with invertase activity [52], which is important for carbon cycling. Our findings imply that *RB41* proliferation likely enhances rhizosphere carbon metabolism, which may be a key microbial mechanism supporting plant adaptations to stress.

Although alpha-diversity indices (Chao1, Shannon, and Simpson) did not differ significantly (Figure S2), species richness and evenness tended to be lower in AS than in CK. By contrast, an analysis of beta-diversity detected a profound restructuring of the microbial community. Both PCoA and PERMANOVA results indicated that stress due to saline–alkaline conditions was the primary driver of community separation, explaining 52.9% of the observed variation (Figure 2, Table S1). This structural shift was accompanied by increased within-group heterogeneity in AS, suggesting that the microbial community may have been more destabilized in AS than in CK under stress conditions. Moreover, differential abundance and LEfSe analyses identified specific taxonomic biomarkers for AS and CK. AS was significantly enriched with various taxa, including the genus *RB41* (Acidobacteriota), Pyrinomonadales, and species belonging to Methylomirabilota as well as several unidentified bacteria (e.g., from Gammaproteobacteria and Thermoleophilia). *RB41* modulates plant health and enhances plant resilience to unfavorable environmental conditions [53,54,55]. These findings collectively demonstrate that saline–alkaline conditions can decrease microbial diversity, while also selecting for a distinct, stress-adapted phylogenetic assemblage and suppressing taxa typically associated with non-stressed conditions.

Predicted microbial gene functions elucidated the metabolic strategies of microbial communities exposed to saline–alkaline conditions. The significant increase in genes encoding oxidative stress-responsive enzymes, including peroxiredoxin and glutathione reductase, in AS may be related to a critical adaptive mechanism that protects against ROS-induced damage under highly saline and alkaline conditions (Figure 4). In addition, the increased abundance of genes encoding chloride channel proteins may be associated with an active ion homeostasis strategy for mitigating chloride toxicity through efflux mechanisms [56]. By contrast, the CK group had a functional profile oriented toward basal maintenance, as evidenced by the enrichment of mannitol dehydrogenase [57] (osmolyte synthesis) and L-rhamnosyltransferase [58] (cell wall reinforcement) genes. These findings imply that in saline–alkaline environments, microbial functional priorities shift from general cellular maintenance to urgent stress mitigation. Notably, the differential abundance of nickel transport-related genes highlights a potentially overlooked aspect of saline–alkaline ecosystems. More specifically, changes in metal ion bioavailability may modulate the trace element acquisition pathways of microorganisms, possibly influencing key metalloenzyme-dependent processes (e.g., nitrogen fixation and antioxidant defense). This functional divergence underscores a systematic reprogramming of microbial metabolism in response to complex saline–alkaline stressors.

Our cultivation-dependent approach successfully isolated 42 microbial strains from saline–alkaline soil. These strains may be useful for validating high-throughput sequencing data and for future biotechnological applications. The dominance of *Bacillus* (47.62%) among culturable microbes is a noteworthy finding, with possible implications for improving soil activities and physicochemical properties of saline–alkaline soils [59,60]. The reported capacity of *P. megaterium* (i.e., species most closely related to strain M29) to enhance the antioxidant scavenging system [61] is consistent with the strong oxidative stress response predicted for the AS microbial community by PICRUSt2 (Figure 4).

All isolated strains were analyzed in terms of their functional traits, leading to the selection of the following six strains: *P. megaterium* M29, *Bacillus* sp. M59, *P. laurentiana* M73, *S. maltophilia* M81, *Stenotrophomonas* sp. M95, and *A. guillouiae* M97. These strains were categorized into three groups: broad-spectrum strains, specialized strains, and intermediate strains. A pot experiment was conducted to validate their PGP effects, which confirmed that the performance of some strains was consistent with their group assignments. *S. maltophilia* M81, which was phylogenetically related to known IAA producers, yielded the most pronounced increase in plant height (Figure 8b). This result is consistent with reports that *S. maltophilia* improves tomato and wheat growth and yield through a mechanism that may be linked to enhanced IAA biosynthesis [62,63]. This provides further evidence that this pathway has positive effects on plant growth. Although *Pseudomonas* species have been confirmed as PGPR, the PGP capacity of *P. laurentiana* (i.e., same species as strain M73) remains uncharacterized, particularly under saline–alkaline conditions [64,65,66]. Thus, *P. laurentiana* M73 represents a novel and promising candidate for the development of microbial inoculants useful in saline–alkaline soils. Furthermore, FH also had significant effects, particularly on fresh weight, underscoring the benefit of combining multiple strains, which likely mirrors the natural microbial support system.

The significant enhancement of SOD and POD activities observed in this study (Figure 9) demonstrates that M73 and FH can activate the antioxidant defense system in alfalfa exposed to saline–alkaline conditions. This finding is consistent with the reported results for other PGPR–plant systems under salt stress conditions. In soybean (*Glycine max* (L.) Merrill), an inoculation with *Bacillus aryabhattai* ALT29 and *Arthrobacter woluwensis* ALT43 can increase the glutathione content, while decreasing lipid peroxidation and superoxide anion levels, reflecting a decrease in the oxidative burden in saline environments [67]. *Enterobacter cloacae* ZNP-4 can increase SOD, catalase (CAT), and POD activities in wheat treated with NaCl, leading to decreased ROS levels [68]. Similarly, *Enterobacter ludwigii* B30 reportedly increases CAT and SOD activities in bermudagrass, which is crucial for mitigating salt-induced oxidative damage [34]. Enhanced antioxidant enzyme activities coupled with a decrease in oxidative damage markers revealed by these studies strongly support the existence of a conserved mechanism: PGPR alleviate the harmful effects of excessive salinity and alkalinity stress on plants at least partly by activating the host enzymatic antioxidant system, thereby maintaining cellular redox homeostasis and protecting membrane integrity. Furthermore, our pot experiment results validated the in vitro functional predictions and clearly demonstrated the capacity of selected PGPR strains to protect alfalfa against saline–alkaline stress, likely through distinct strain-specific mechanisms. Future research should focus on field trials to validate these greenhouse findings and explore the synergistic effects of bacterial consortia. Metagenomic and transcriptomic analyses may further elucidate the precise molecular mechanisms underlying this beneficial partnership.

## 4. Materials and Methods

### 4.1. Soil Sampling and Site Description

Soil samples were collected from the rhizosphere of 2-year-old alfalfa (*M. sativa* L. cv. ‘Nongjing 1’) plants at the full flowering stage. AS samples were characterized by substantial amounts of Na_2_CO_3_/NaHCO_3_. CK samples comprised phaeozem soil. Sampling sites were in Harbin, Heilongjiang Province, China (46°31′22.86″ N to 46°31′24.00″ N, 125°28′36.85″ E to 125°28′50.52″ E). This region has a semi-arid continental climate with a mean annual temperature of 3.2 °C and a mean annual precipitation of approximately 550 mm. Three independent biological replicates were prepared per soil type. For each replicate, rhizosphere soil from 10 individual plants within a designated plot was collected and combined to form a single composite sample. This sampling strategy resulted in a total of six composite samples (three for AS and three for CK).

For DNA extractions and 16S rRNA gene sequencing, the six composite samples were processed individually as independent biological replicates. A portion of each was sub-packaged and stored at −80 °C. To isolate bacteria, soil from three AS composite samples was combined to create one mixed AS sample, which was then temporarily stored at 4 °C. For an analysis of soil physicochemical properties, a separate set of samples was created. Soil from three composite samples of the same type (AS or CK) was combined and mixed to form one representative mixed sample per soil type. This mixed sample was then air-dried, passed through a 2 mm sieve, and analyzed (see Table 1 for results).

### 4.2. Soil Physicochemical Analysis

Soil pH of AS and CK was determined using the potentiometric method with a soil-to-water ratio of 1:2.5. The SOM content was measured according to a potassium dichromate oxidation–external heating method [69]. Total nitrogen content was determined using a semi-micro Kjeldahl method [70] and hydrolyzable nitrogen content was analyzed using an alkali-hydrolysis diffusion method [71]. Total phosphorus content was determined using a sodium hydroxide fusion–molybdenum–antimony anti-colorimetric method [72]. Available phosphorus content was determined according to a sodium bicarbonate extraction–molybdenum–antimony anti-colorimetric method [73]. Total potassium and available potassium content was measured via flame photometry after extractions using sodium hydroxide and ammonium acetate, respectively [70]. All soil physicochemical properties were analyzed at the Heilongjiang Institute of Black Soil Protection and Utilization.

### 4.3. 16. S rRNA Gene Sequencing and Bioinformatic Analysis

Total genomic DNA was extracted from 0.25 g rhizosphere soil samples using a Magnetic Bead Soil DNA Extraction Kit (Tiangen, Beijing, China). The V3–V4 hypervariable region of the bacterial 16S rRNA gene was amplified using primers 338F/806R, which were synthesized by Novogene Co., Ltd. (Beijing, China). The PCR system contained Phusion High-Fidelity DNA Polymerase (New England Biolabs, Ipswich, MA, USA) and 10 ng template DNA. Thermal cycling conditions were as follows: initial denaturation at 98 °C for 1 min; 30 cycles of denaturation at 98 °C for 10 s, annealing at 50 °C for 30 s, and extension at 72 °C for 30 s; final extension at 72 °C for 5 min. Amplicons were verified by gel electrophoresis, after which sequencing libraries were prepared using an NEB Next Ultra DNA Library Prep Kit (New England Biolabs, Ipswich, MA, USA). After a quantification step involving Qubit, libraries were sequenced using an Illumina NovaSeq 6000 platform by Novogene Co., Ltd. (Beijing, China) to generate 250 bp paired-end reads. Raw sequences were processed using FLASH (v1.2.11) for merging and fastp (v0.23.1) for quality control (Q20, length > 200 bp). Chimeric sequences were removed using the SILVA 138.1 database and UCHIME.

A bioinformatic analysis was conducted using QIIME 2 (v2022.2). Within this pipeline, amplicon sequence variants (ASVs) were generated using the DADA2 plugin. Briefly, forward and reverse reads were truncated to 240 and 200 bp, respectively, on the basis of a quality profile inspection to remove low-quality bases. Taxonomic assignments, which were based on the SILVA 138.1 database, were completed using a naive Bayes classifier trained on the 515F/806R region. The resulting feature table was rarefied to 30,000 sequences per sample for all downstream analyses to ensure even sampling depth.

Alpha-diversity was assessed using a suite of indices (Chao1, Shannon and Simpson) calculated by QIIME 2. Beta-diversity was analyzed on the basis of Bray–Curtis dissimilarity. Results were visualized via PCoA. The significance of the differences in community structures between groups was assessed using PERMANOVA (ADONIS), with 999 permutations, in the R vegan package. Differences in features between groups were identified using LEfSe (v1.0), with an LDA score threshold of >2.0. Our overall approach was informed by established bioinformatic pipelines for microbial ecology [74,75,76,77].

### 4.4. Bacterial Isolation and Identification

Bacteria were isolated from 1 g soil (AS and CK) via serial dilution plating on R2A, 0.1× TSA, and 0.1× LB agar media at 28 °C for 2–5 days. Purified isolates were preserved and genomic DNA was extracted using a TIANamp Bacteria DNA Kit (Tiangen Biochemical Technology (Beijing) Co., Ltd., Beijing, China). The 16S rRNA gene was amplified by PCR using primers 27F/1492R (synthesized by BGI, Shenzhen, China) with the following program: 94 °C for 3 min; 32 cycles of 94 °C for 30 s, 53.1 °C for 30 s, and 72 °C for 1.5 min; final extension at 72 °C for 1.5 min. Sequences (with >99% similarity as determined by BLASTn against the NCBI nt database (accessed on [2024-11-24])) were analyzed to reveal phylogenetic relationships, with a neighbor-joining phylogenetic tree constructed using MEGA7 software (1000 bootstrap replicates).

### 4.5. Functional Characterization of Bacterial Strains

Strains were functionally characterized according to the following: saline–alkaline tolerance (using LB medium containing 50, 100, or 200 mM NaHCO_3_ and LB medium containing 300, 500, 700, or 1000 mM NaCl), nitrogen fixation (using Ashby nitrogen-free medium and the indophenol blue method for quantification) [78], phosphate solubilization (using the molybdenum–antimony anti-colorimetric method) [79], and IAA secretion (using King’s medium and the Salkowski colorimetric method) [80]. All analyses were performed using three independent biological replicates. Ammonium nitrogen, available phosphorus, and IAA content was quantitatively analyzed using a spectrophotometer (530 nm/700 nm), with their specific concentrations calculated using standard curves. Details regarding the procedures for determining saline–alkaline tolerance, nitrogen fixation, phosphate solubilization, and IAA secretion are provided in the legends of Figure S4–S9. Functional strains were screened at 28 °C, and all strains were finally stored at −80 °C for later use.

Strain stress resistance characteristics and nitrogen fixation, phosphate solubilization, and IAA production data were standardized using the following formula:

$$Normalized\;value\;=\;\frac{X_{i\;}-\;X_{min}}{X_{max}\;-\;X_{min}}\;\times \;100,$$

where *X_i_* = measured value for strain *i*.

Following the comprehensive evaluation of all strains, six strains ranked among the top 10 in terms of the analyzed parameters were selected. Data were visualized as radar charts using R (v4.3.2) with the fmsb package (v0.7.5). Selected strains were cryopreserved at −80 °C for downstream applications.

### 4.6. Screening of PGPR Strains and Pot Experiments

Experiments were conducted using excellent PGPR strains, including single strains and FH screened from the rhizosphere of alfalfa grown in saline–alkaline soil. PGPR strains were activated on LB solid medium. For each strain, a single colony was used to inoculate LB liquid medium. Cultures were incubated at 28 °C for 12 h with shaking (200 rpm). Bacterial cells were collected by centrifugation at 5000 rpm, washed twice with sterile water, and finally resuspended in PBS buffer. Bacterial suspensions were adjusted to OD_600_ = 1. The FH suspension was prepared by mixing equal volumes of each bacterial suspension (adjusted to OD_600_ = 1 immediately before mixing to ensure a standardized and comparable initial contribution from each strain).

After alfalfa seeds were soaked in a bacterial suspension for 2 h, a germination test was conducted in the presence of 0, 30, or 50 mM NaHCO_3_. Thirty seeds were placed in each Petri dish, with three replicates per treatment. Petri dishes were incubated in a growth chamber set at 25 °C, 70% relative humidity, and a 16 h light/8 h dark photoperiod. The germination rate was recorded and calculated on day 7.

For pot experiments, sterilized vermiculite–peat soil (1:1) was used as the substrate. Surface-sterilized seeds were germinated on half-strength MS medium and then uniformly growing seedlings at the first true-leaf stage were selected for transplanting (three seedlings per pot). The experiment included a total of nine treatments, each with three replicates. Plants were grown in a controlled-environment chamber with a 16 h light/8 h dark photoperiod. After an acclimation period with regular watering, the stress treatment was initiated. Two control groups were established: ck1 plants received 50 mL sterile water every 5 days, whereas ck2 plants received 50 mL 50 mM NaHCO_3_ solution every 5 days. Plants in the seven treatment groups received 50 mL 50 mM NaHCO_3_ solution on the same 5-day schedule.

For bacterial inoculations, all groups were treated every 3 days with 6 mL bacterial suspension or sterile water. Treatment groups were inoculated with 6 mL bacterial suspension comprising single strains or FH. Control plants (ck1 and ck2) were treated with 6 mL sterile water instead of a bacterial suspension. After 55 days, plant growth indices (height, root length, fresh weight, and dry weight) and physiological indices (total chlorophyll content [81,82], soluble protein content [83], and SOD and POD activities) were determined. SOD (WST-1 method) and POD activities were measured using commercial assay kits from Solarbio Science & Technology Co., Ltd. (Beijing, China). All experiments were completed using three independent biological replicates.

## 5. Conclusions

**Figure 10:**
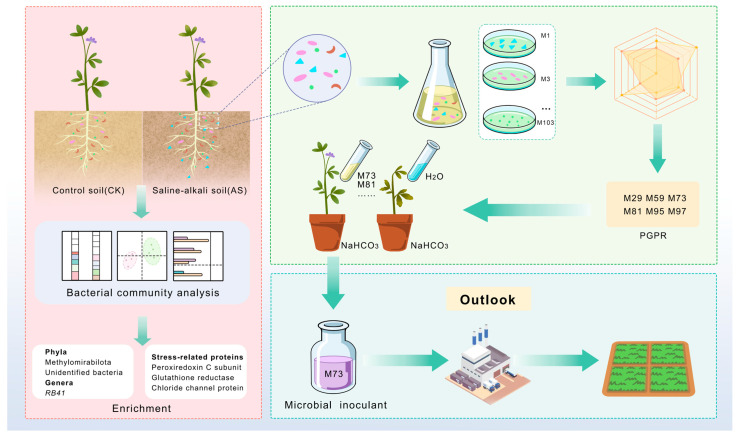
Overview of the isolation, validation, and potential utility of PGPR from the alfalfa rhizosphere in saline–alkaline soil.

This study demonstrates that the alfalfa rhizosphere microbial community undergoes significant structural shifts in saline–alkaline soil (i.e., significant changes in beta-diversity). These shifts are accompanied by observable changes in microbial community compositions at the phylum and genus levels. Predicted microbial functions revealed a significant enrichment of genes encoding proteins related to oxidative stress responses in saline–alkaline soil. Subsequently, we isolated 42 culturable strains, among which six (*P. megaterium* M29, *Bacillus* sp. M59, *P. laurentiana* M73, *S. maltophilia* M81, *Stenotrophomonas* sp. M95, and *A. guillouiae* M97) were selected for pot experiments on the basis of an integrated analysis of their PGP traits. Finally, strains M73 and M81 were observed to significantly enhance alfalfa seed germination, plant growth, biomass accumulation, and physiological and antioxidant parameters under stress conditions due to the presence of NaHCO_3_. Notably, the PGP effect of *P. laurentiana* M73 has not been reported previously (Figure 10). The PGP effects of these strains will need to be more systematically evaluated in field trials. Furthermore, considering the potential utility of selected strains for protecting plants against excessive NaHCO_3_ concentrations, future investigations should assess their performance and synergistic mechanisms under natural saline–alkaline conditions.

## Figures and Tables

**Table 1:** Soil physicochemical properties.

	TN ^#^ g/kg	TP g/kg	TK g/kg	AN mg/kg	AP mg/kg	AK mg/kg	SOM g/kg	pH
CK	2.10	0.81	17.70	136.82	24.25	149.10	34.80	7.88
AS	3.20	0.74	23.00	224.92	16.95	157.40	53.90	8.25

^#^ TN, TP, TK, AN, AP, AK, and SOM represent total nitrogen, total phosphorus, total potassium, available nitrogen, available phosphorus, available potassium, and soil organic matter, respectively.

**Table 2:** Taxonomic identification of dominant culturable isolates.

Strain ID	Comparative Information	Phylogenetic Affiliation	Similarity (%)	GenBank
M1	*Bacillus* sp.	*Bacillus*	98.94%	OQ876139.1
M2	*Enterobacter mori*	*Enterobacter*	94.70%	LC617171.1
M3	*Proteus mirabilis*	*Proteus*	99.65%	OL629224.1
M5	*Pseudomonas lini*	*Pseudomonas*	100.00%	OQ654023.1
M7	*Escherichia* sp.	*Escherichia*	99.07%	KJ803863.1
M9	*Escherichia coli*	*Escherichia*	99.58%	MN704526.1
M10	*Bacillus siamensis*	*Bacillus*	99.93%	PV012710.1
M13	*Janthinobacterium svalbardensis*	*Janthinobacterium*	99.22%	MW927167.1
M16	*Bacillus* sp.	*Bacillus*	100.00%	HQ433576.1
M17	*Novosphingobium barchaimii*	*Novosphingobium*	100.00%	MW433633.1
M21	*Duganella zoogloeoides*	*Duganella*	99.29%	MN752691.1
M22	*Bacillus* sp.	*Bacillus*	99.86%	KC119103.1
M25	*Brevundimonas* sp.	*Brevundimonas*	100%	MK414927.1
M27	*Bacillus cereus*	*Bacillus*	99.93%	MG027629.1
M29	*Bacillus* sp.	*Bacillus*	99.86%	KT900618.1
M32	*Bacillus* sp.	*Bacillus*	100.00%	PQ657652.1
M33	*Bacillus toyonensis*	*Bacillus*	99.93%	MW405814.1
M36	*Achromobacter xylosoxidans*	*Achromobacter*	99.79%	KJ569364.1
M39	*Peribacillus frigoritolerans*	*Peribacillus*	99.59%	OM281797.1
M40	*Bacillus* sp.	*Bacillus*	99.86%	MW116732.1
M49	*Bacillus* sp.	*Bacillus*	99.93%	MW753132.1
M51	*Bacillus* sp.	*Bacillus*	100.00%	MH329935.1
M53	*Paenibacillus* sp.	*Paenibacillus*	99.58%	AM162308.1
M59	*Bacillus* sp.	*Bacillus*	100.00%	KT583425.1
M62	*Bacillus* sp.	*Priestia*	98.21%	OM346694.1
M64	*Bacillus acidiceler*	*Bacillus*	99.79%	KJ575070.1
M66	*Stenotrophomonas* sp.	*Stenotrophomonas*	99.93%	OP765271.1
M67	*Enterobacter* sp.	*Enterobacter*	99.72%	KJ584024.1
M68	*Pseudomonas chlororaphis*	*Pseudomonas*	100.00%	OQ363217.1
M69	*Neobacillus* sp.	*Neobacillus*	99.79%	OR878890.1
M72	*Bacillus thuringiensis*	*Bacillus*	100.00%	OP986100.1
M73	*Pseudomonas putida*	*Pseudomonas*	99.93%	HM486417.1
M77	*Kosakonia oryzendophytica*	*Kosakonia*	99.65%	MW020337.1
M79	*Pseudomonas protegens*	*Pseudomonas*	100.00%	PQ573341.1
M81	*Stenotrophomonas maltophilia*	*Stenotrophomonas*	99.93%	JQ659631.1
M89	*Bacillus* sp.	*Bacillus*	100.00%	OR362817.1
M92	*Bacillus* sp.	*Bacillus*	97.81%	MN044783.1
M93	*Escherichia* sp.	*Escherichia*	99.30%	OQ876054.1
M95	*Stenotrophomonas geniculata*	*Stenotrophomonas*	100.00%	KJ452162.2
M97	*Acinetobacter guillouiae*	*Acinetobacter*	100.00%	MH144279.1
M98	*Peribacillus frigoritolerans*	*Peribacillus*	100.00%	MZ712051.1
M103	*Kosakonia oryzendophytica*	*Kosakonia*	99.79%	PQ781316.1

## Data Availability

Raw data supporting the conclusions of this article will be made available by the authors on request.

## Supplementary Materials

The following supporting information can be downloaded at: https://www.mdpi.com/article/10.3390/plants14243844/s1, Figure S1: Relative abundance of the dominant bacterial genera in saline–alkaline soil (AS) and control soil (CK); Figure S2: Microbial alpha-diversity of alfalfa rhizosphere soil under saline–alkaline conditions; Figure S3: T-test of intergroup species differences; Figure S4: Growth of strains treated with different NaHCO_3_ concentrations; Figure S5: Growth of strains treated with different NaCl concentrations; Figure S6: Nitrogen-fixing capacity of strains; Figure S7: Determination of ammonium nitrogen contents secreted by strains; Figure S8: Phosphate-solubilizing capacity of bacterial strains in inorganic phosphorus medium (a) and organic phosphorus medium (b); Figure S9: IAA-producing capacity of strains; Table S1: PERMANOVA results for the effects of saline–alkaline soil conditions on the microbial community structure; Table S2: Normalized values for the function-related traits of 42 PGPR strains (%).

## Abbreviations

The following abbreviations are used in this manuscript:

PGPR	Plant growth-promoting rhizobacteria
AS	Saline–alkaline soil
CK	Control soil
PGP	Plant growth-promoting
ROS	Reactive oxygen species
TN	Total nitrogen
TP	Total phosphorus
TK	Total potassium
AN	Available nitrogen
AP	Available phosphorus
AK	Available potassium
SOM	Soil organic matter
LEfSe	Linear discriminant analysis Effect Size
PCoA	Principal Co-ordinates Analysis
IAA	Indole-3-acetic acid
OTUs	Operational taxonomic units
SOD	Superoxide dismutase
POD	Peroxidase
CAT	Catalase
R2A	Reasoner’s 2A agar
TSA	Tryptic Soy Agar
LB	Lysogeny Broth
ASVs	Amplicon Sequence Variants
