# Supplementary material for: Saline–Alkaline Stress-Driven Rhizobacterial Community Restructuring and Alleviation of Stress by Indigenous PGPR in Alfalfa
Source: Plants (Basel). 2025 Dec 17;14(24):3844. doi: 10.3390/plants14243844 (PMC12737148; doi:10.3390/plants14243844)
Supplement: Supplementary file 1 [file plants-14-03844-s001.zip › plants-4012911-supplementary.pdf]

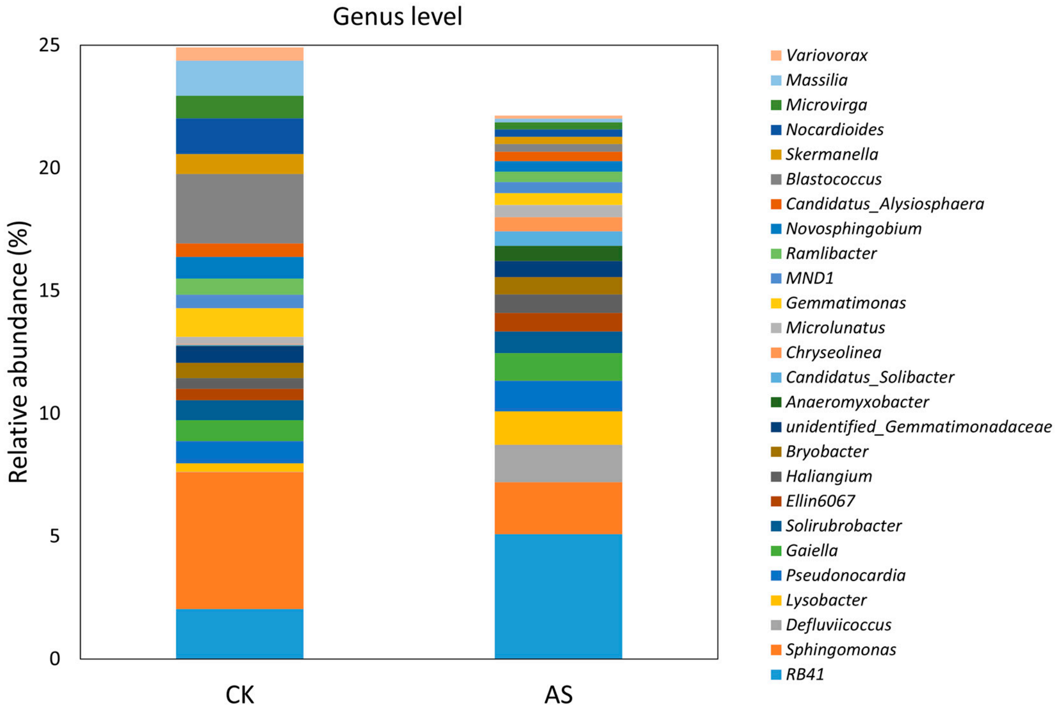

Figure S1. Relative abundance of the dominant bacterial genera in saline-alkaline soil (AS) and control soil (CK). This figure details the bacterial community composition presented in the main text's Figure 1c, but exclusively displays genera with a relative abundance of ≥ 0.5%, with the "Others" category omitted for clarity. The data are derived from three independent biological replicates (n=3). Genera are sorted by their mean relative abundance across all replicates.

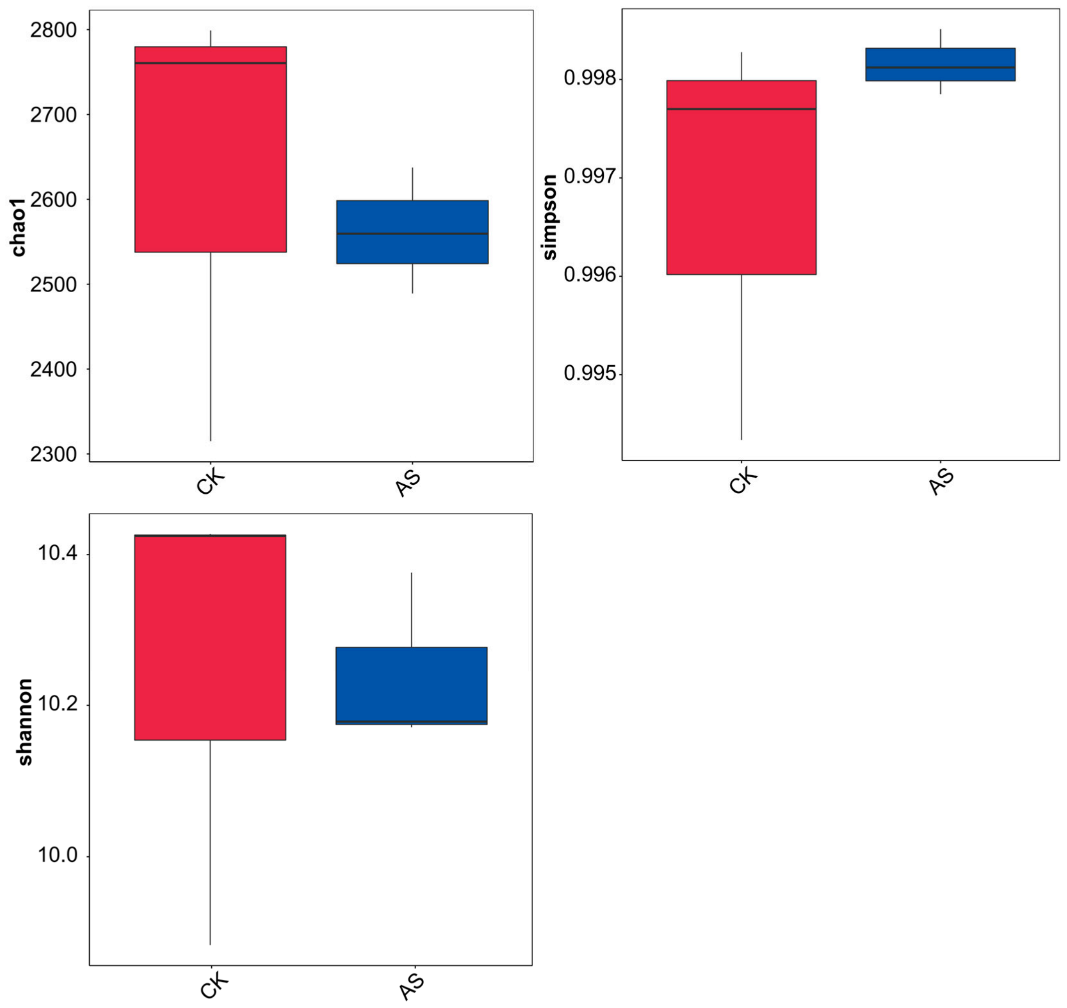

Figure S2. Microbial alpha-diversity of alfalfa rhizosphere soil under saline-alkaline conditions; definitions persist in all analyses.

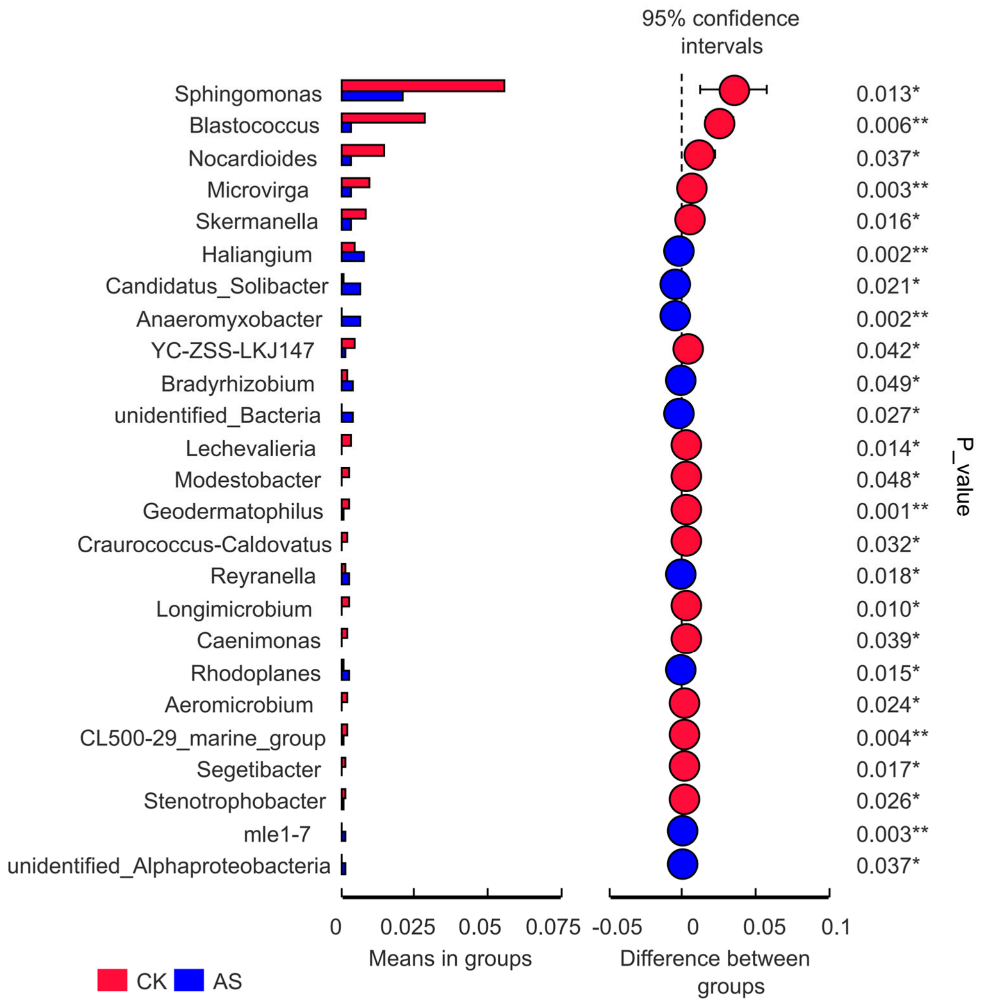

Figure S3. *T*-test of intergroup species differences (Left) Mean abundance of differential species. (Right) Mean differences (95% CI; color: *p*-value). Abbreviations as previously defined.

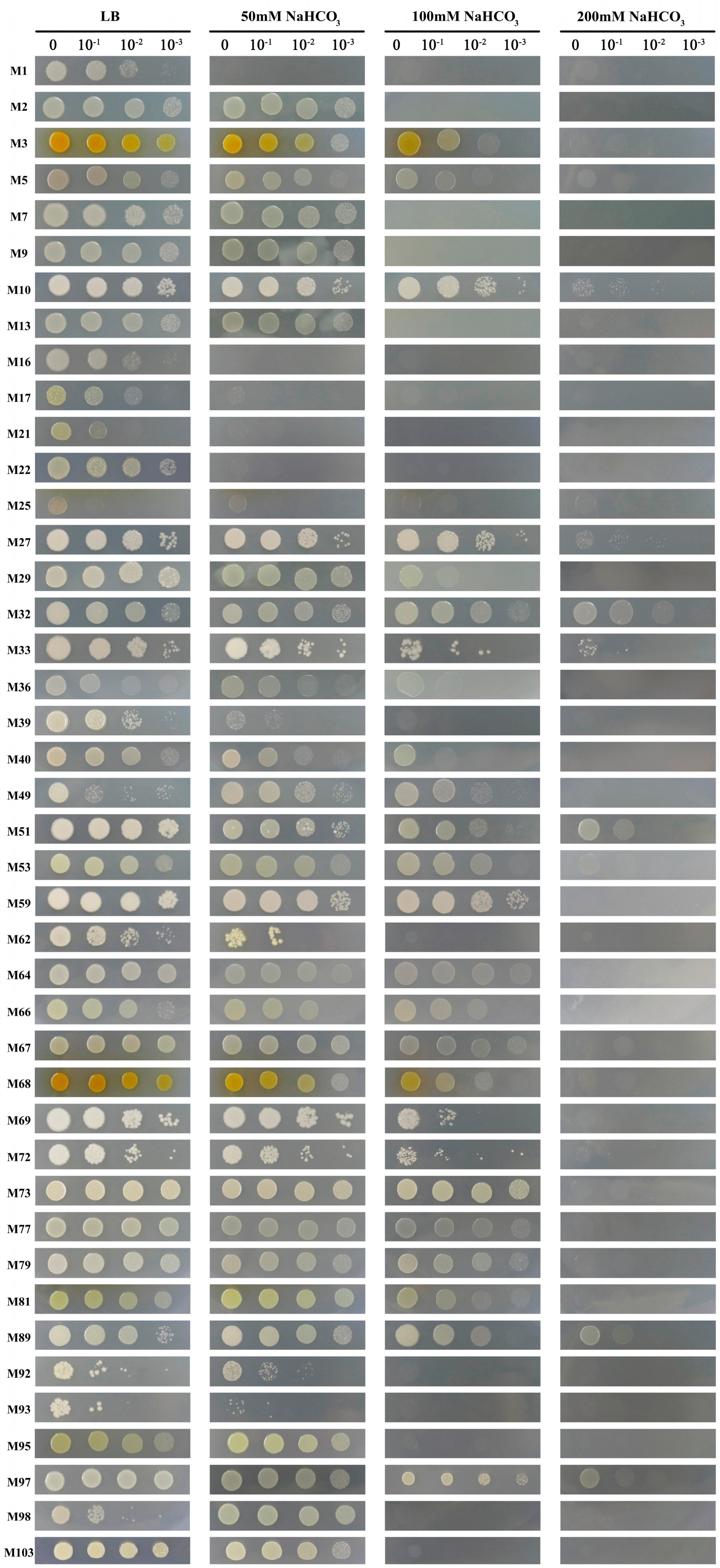
LB
50mM NaHCO3
100mM NaHCO3
200mM NaHCO3
0 10-1 10-2 10-3
M1
M2
M3
M5
M7
M9
M10
M13
M16
M17
M21
M22
M25
M27
M29
M32
M33
M36
M39
M40
M49
M51
M53
M59
M62
M64
M66
M67
M68
M69
M72
M73
M77
M79
M81
M89
M92
M93
M95
M97
M98
M103

Figure S4. Growth of strains treated with different $NaHCO_3$ concentrations. The optical density at 600 nm (OD_{600}) of all bacterial strains was adjusted to 1.0 using spectrophotometry. The bacterial suspensions were serially diluted from 10^{-1} to 10^{-3}. A 10 μL aliquot from each dilution was spot-inoculated onto LB solid media containing $NaHCO_3$ at concentrations of 50, 100 and 200 mM. The plates were incubated statically at 30 °C for 1-2 days, after which the growth status of the strains was observed. The growth of strains on standard LB medium served as the control.

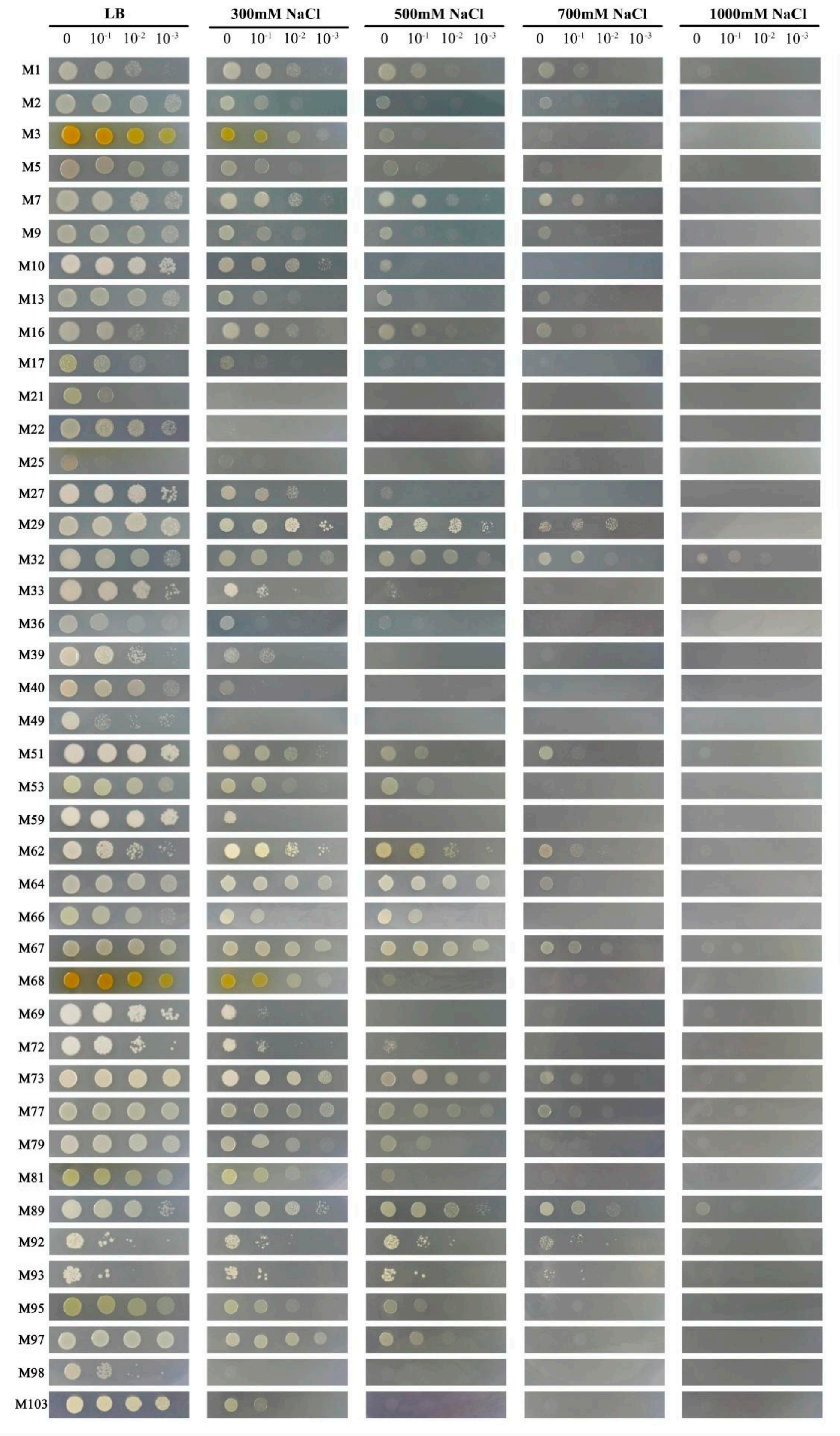

Figure S5. Growth of strains treated with different NaCl concentrations. The optical density at 600 nm (OD_{600}) of all bacterial strains was adjusted to 1.0 using spectrophotometry. The bacterial suspensions were serially diluted from 10^{-1} to 10^{-3}. A 10 μL aliquot from each dilution

was spot-inoculated onto LB solid media containing NaCl at concentrations of 300, 500, 700 and 1000 mM. The plates were incubated statically at 30 ℃ for 1-2 days, after which the growth status of the strains was observed. The growth of strains on standard LB medium served as the control.

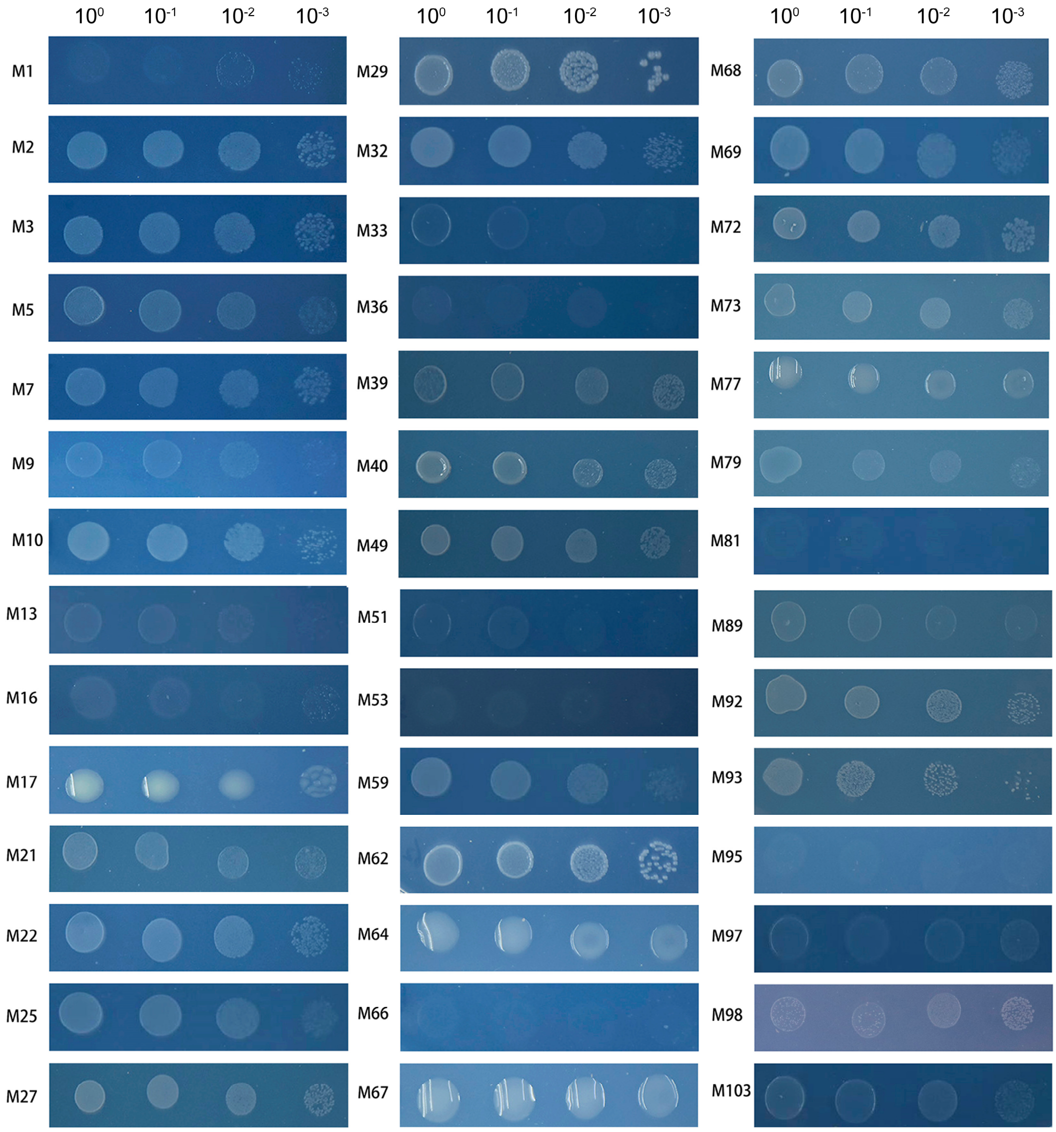

Figure S6. Nitrogen-fixing capacity of strains. The OD_{600} of all bacterial strains was adjusted to 0.5 using a spectrophotometer. The bacterial suspensions were serially diluted from 10^{-1} to 10^{-3}. A 4 μL aliquot from each dilution (100 to 10^{-3}) was inoculated vertically onto Ashby nitrogen-free solid medium. The plates were incubated inverted at 28°C for 2-5 days. The growth of strains on the nitrogen-free medium was observed to preliminarily assess their nitrogen-fixing ability.

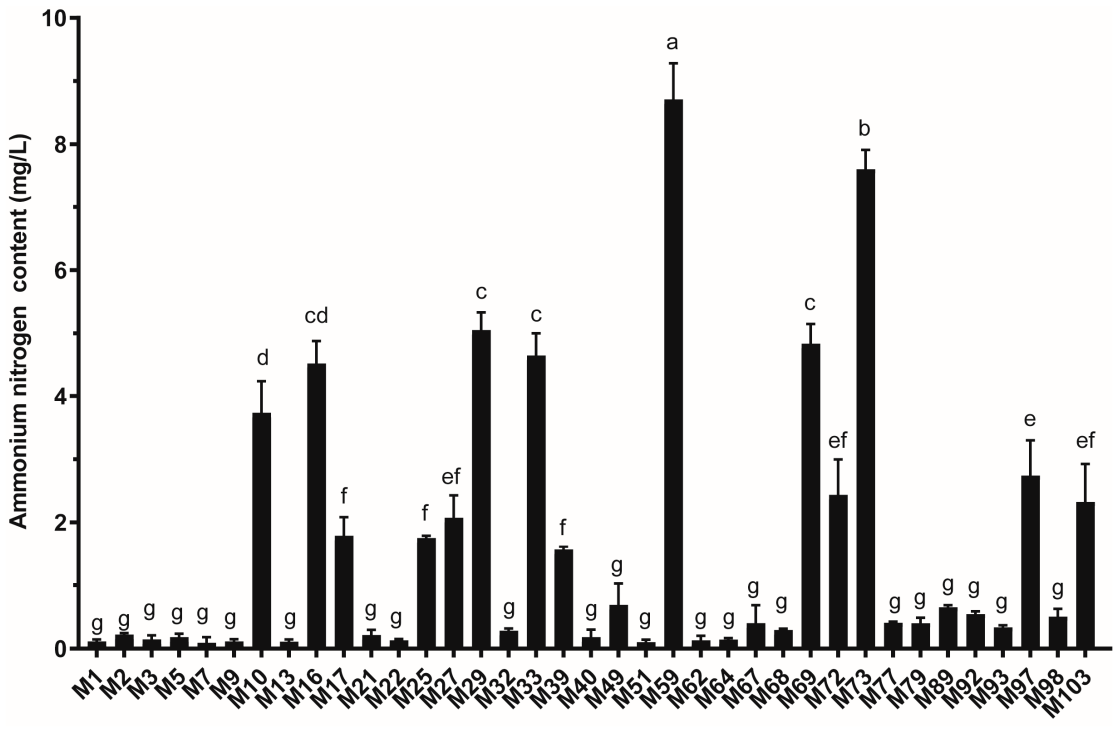

Figure S7. Determination of ammonium nitrogen contents secreted by strains. The quantitative determination of nitrogen-fixing capacity was performed using the indophenol blue-spectrophotometric method. The specific procedure was as follows: Activated bacteria were centrifuged to obtain the cell pellet. The pellet was washed with Ashby nitrogen-free liquid medium to remove any residual LB medium. Subsequently, 0.5 g of the washed bacterial cells was inoculated into 50 mL of Ashby nitrogen-free liquid medium and cultured at 28°C with shaking at 200 rpm for 24 hours. A 2 mL aliquot of the bacterial culture was transferred to a new centrifuge tube and centrifuged at 6000 rpm for 10 minutes. Then, 100 μL of the supernatant was pipetted into a 15 mL centrifuge tube, serving as the test sample. To this sample, 5 mL of Solution A and 5 mL of Solution B were added sequentially. The mixture was vortexed thoroughly and then subjected to a color development reaction in a 37°C water bath for 20 minutes. After cooling, the absorbance at a wavelength of 530 nm was measured using a UV spectrophotometer. The ammonium nitrogen content secreted by the bacterial strain was calculated based on the standard curve for ammonium nitrogen. All data is represented as mean ± SD, with different letters indicating significant differences (p<0.05).

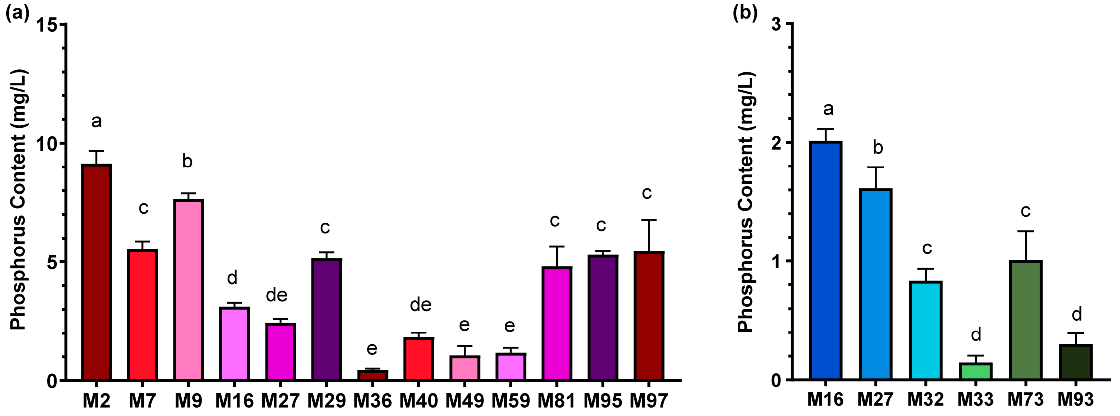

Figure S8. Phosphate-solubilizing capacity of bacterial strains in inorganic phosphorus medium (a) and organic phosphorus medium (b). Quantitative determination was performed using the molybdenum-antimony anti-spectrophotometric method. The specific procedure was as follows: Bacterial suspensions were prepared and inoculated at a 1% (v/v) inoculum size into Pikovskaya's inorganic phosphorus liquid medium and Pikovskaya's organic phosphorus liquid medium, respectively. Each treatment was performed in triplicate, with uninoculated medium serving as the control. The cultures were incubated on a shaker at 28°C and 160 rpm for 12 days. After incubation, the pH value of each bacterial culture was measured. A 10 mL aliquot of the culture was centrifuged at 8000 rpm and 4°C for 15 minutes. Then, 5 mL of the supernatant was transferred to a 150 mL conical flask. To this, 45 mL of 0.5 mol/L $NaHCO_3$ extraction solution was added, followed by a spoonful of phosphorus-free activated carbon powder. The flask was stoppered tightly and shaken on a rotary shaker for 30 minutes, then immediately filtered through phosphorus-free filter paper. A 10 mL aliquot of the filtrate was pipetted into a 50 mL volumetric flask, followed by the addition of 30 mL of distilled water and 5 mL of the molybdenum-antimony anti-color developing agent. The volume was made up to 50 mL with distilled water, and the solution was mixed thoroughly. After standing for 30 minutes, colorimetry was performed at a wavelength of 700 nm. The absorbance of the developed solution was recorded, and the phosphorus concentration (μg/mL) was determined from the standard curve. The value of available phosphorus increment (μg/mL) was calculated by subtracting the value of the control. All data is represented as mean ± SD, with different letters indicating significant differences ($p<0.05$).

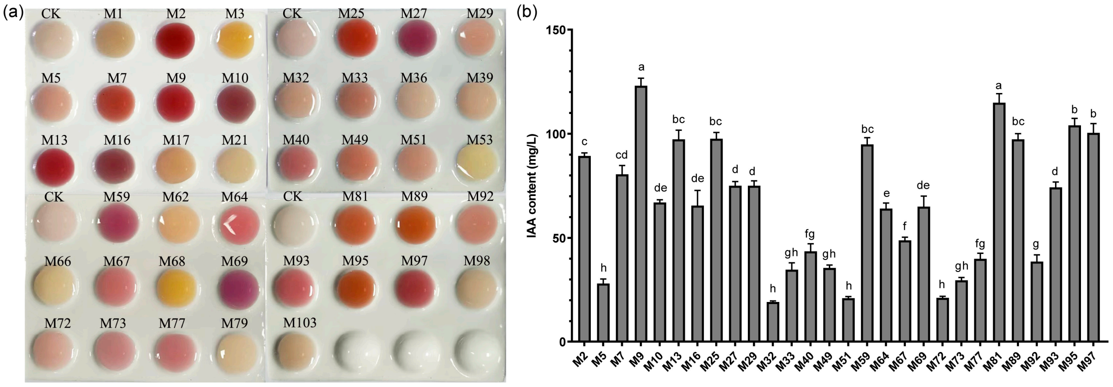

Figure S9. IAA-producing capacity of strains. (a) Qualitative analysis of IAA production by the isolated strains. 50 mL of liquid King's medium was placed in a 150 mL Erlenmeyer flask and sterilized at 121 °C for 30 minutes. After cooling, 500 μL of each bacterial suspension (10^8 cfu/mL) was inoculated into the flasks. Each strain was tested in triplicate, with uninoculated medium serving as the control (CK). The flasks were incubated in a shaker at 28°C and 150 rpm for 12 days. A 50 μL aliquot of the bacterial suspension was dropped onto a white ceramic plate, followed immediately by the addition of 50 μL of Salkowski's reagent. For the control, 50 μL of 10 ppm IAA standard solution was added to 50 μL of Salkowski's reagent instead of the culture supernatant. The ceramic plate was kept at room temperature, and color development was observed within 15 minutes. A color change to pink indicated the ability to secrete IAA, with a deeper color suggesting a stronger IAA secretion ability. No color change was considered negative, indicating no IAA secretion. (b) Quantitative analysis of IAA production by the isolated strains. The aforementioned culture broth was centrifuged at 10000 rpm and 4 °C for 10 minutes. A 5 mL aliquot of the supernatant was mixed with 5 mL of Salkowski's reagent (S2). The mixture was allowed to stand in the dark for 30 minutes. Subsequently, the absorbance of the test solution was measured immediately at a wavelength of 530 nm using a spectrophotometer. The IAA concentration (μg/mL) of the test solution was determined from the standard curve. All data is represented as mean ± SD, with different letters indicating significant differences (p<0.05).

Table S1. PERMANOVA results for the effects of saline-alkaline soil conditions on the microbial community structure

	Df	SumsOfSqs	MeanSqs	F.Model	R^2	Pr(>F)
**Description**	1	0.728884	0.728884	4.483689	0.528507	0.1
**Residuals**	4	0.650254	0.162563	NaN	0.471493	NaN
**Total**	5	1.379138	NaN	NaN	1	NaN

Table S2. Normalized values for the function-related traits of 42 PGPR strains (%)

Strains ID	Saline-alkali tolerance	Nitrogen fixation	P solubilization	IAA production
M1	11.76470588	0.218755657	NA	NA
M2	29.41176471	1.487074268	100	67.54671832
M3	35.29411765	0.612283739	NA	NA
M5	35.29411765	1.027861463	NA	8.529598062
M7	41.17647059	0	59.8209584	59.01707518
M9	29.41176471	0.218755657	83.35966298	100
M10	58.82352941	42.22610863	NA	46.01952411
M13	17.64705882	0.175004526	NA	75.22337888
M16	11.76470588	51.38866323	55.39757767	44.55727567
M17	0	19.63706289	NA	NA
M21	NA	1.421389545	NA	NA
M22	NA	0.459212805	NA	NA
M25	NA	19.24341876	NA	75.50774053
M27	58.82352941	22.93905479	43.44391784	53.77737005
M29	41.17647059	57.53343422	55.55555555	53.77737326
M32	100	2.18674422	7.635597684	0
M33	35.29411765	52.83187032	0	14.94718035
M36	17.64705882	NA	3.422854136	NA
M39	NA	17.05667454	NA	NA
M40	0	1.005927872	18.7467088	23.43625159
M49	17.64705882	6.932059251	10.16324381	15.75954595
M51	35.29411765	0.087502263	NA	1.7465042
M53	35.29411765	NA	NA	NA
M59	17.64705882	100	11.47972618	72.86762455
M62	23.52941176	0.459212805	NA	NA
M64	47.05882353	0.546715068	NA	43.17628304
M66	17.64705882	3.564382633	NA	NA
M67	58.82352941	2.317997614	NA	28.51336808
M68	35.29411765	NA	NA	NA

M69	0	55.01861454	NA	44.10570475
M72	17.64705882	27.18128983	NA	1.949619664
M73	58.82352941	87.16385901	9.531332283	10.03245997
M77	58.82352941	3.651884896	NA	19.98377002
M79	47.05882353	3.564382633	NA	NA
M81	35.29411765	NA	51.76408637	92.12027516
M89	58.82352941	6.516597578	NA	75.22337888
M92	41.17647059	5.226345376	NA	18.68402357
M93	NA	2.82096155	1.737756717	53.00572381
M95	29.41176471	NA	57.18799369	81.68158426
M97	70.58823529	30.72385493	58.92575039	78.1884796
M98	0	4.789066163	NA	NA
M103	0	25.89115368	NA	NA
